# Hospital Sunday and the Queen's Jubilee

**Published:** 1887-06-04

**Authors:** 


### HOSPITAL SUNDAY AND THE QUEEN'S JUBILEE.

Mr. John Libby, Hon. Sec. of the Stroud Hospital, writes as follows: "The 19th June is the first day of the Jubilee Week, and it happens also to be 'Hospital Sunday.' What more appropriate object for our thank-offerings than the hospitals throughout the country? If the press would take it up, I am sure a hearty response from 'ministers of all denominations' would be the result. How could we better begin the Jubilee Week than by a simultaneous effort to help the sick and needy? Only think, Sir, of the sympathy which such an act of charity would bring out; and how gratified the Queen herself would be at such a manifestation of the nation's gratitude. To give currency to the idea, may I ask you to insert this letter in the next issue of The Hospital, and use your influence to get it reproduced in the daily press? For the last ten years the third Sunday in June has been Hospital Sunday in Stroud, and we hope this year's collection will exceed all others, good as they always have been, as a special effort will be made to advocate the claims of our institution by identifying it with the Queen's Jubilee."

